# Role of Bacterial Community Composition as a Driver of the Small-Sized Phytoplankton Community Structure in a Productive Coastal System

**DOI:** 10.1007/s00248-022-02125-2

**Published:** 2022-10-28

**Authors:** Cecilia Costas-Selas, Sandra Martínez-García, Ramiro Logares, Marta Hernández-Ruiz, Eva Teira

**Affiliations:** 1 Centro de Investigación Mariña, Universidade de Vigo, Departamento de Ecoloxía e Bioloxía Animal, 36310 Vigo, Spain; 2 Departament de Biologia Marina I Oceanografia, Institut de Ciéncies del Mar (ICM), CSIC, Catalonia Barcelona, Spain

**Keywords:** Productive system, Phytoplankton-bacteria interactions, 18S rRNA gene, 16S rRNA gene, Bacterial seasonality, Microbial communities

## Abstract

We present here the first detailed description of the seasonal patterns in bacterial community composition (BCC) in shelf waters off the Ría de Vigo (Spain), based on monthly samplings during 2 years. Moreover, we studied the relationship between bacterial and small-sized eukaryotic community composition to identify potential biotic interactions among components of these two communities. Bacterial operational taxonomic unit (OTU) richness and diversity systematically peaked in autumn–winter, likely related to low resource availability during this period. BCC showed seasonal and vertical patterns, with *Rhodobacteraceae* and *Flavobacteriaceae* families dominating in surface waters, and SAR11 clade dominating at the base of the photic zone (30 m depth). BCC variability was significantly explained by environmental variables (e.g., temperature of water, solar radiation, or dissolved organic matter). Interestingly, a strong and significant correlation was found between BCC and small-sized eukaryotic community composition (ECC), which suggests that biotic interactions may play a major role as structuring factors of the microbial plankton in this productive area. In addition, co-occurrence network analyses revealed strong and significant, mostly positive, associations between bacteria and small-sized phytoplankton. Positive associations likely result from mutualistic relationships (e.g., between *Dinophyceae* and *Rhodobacteraceae*), while some negative correlations suggest antagonistic interactions (e.g., between *Pseudo-nitzchia* sp. and SAR11). These results support the key role of biotic interactions as structuring factors of the small-sized eukaryotic community, mostly driven by positive associations between small-sized phytoplankton and bacteria.

**Supplementary Information:**

The online version contains supplementary material available at 10.1007/s00248-022-02125-2.

## Introduction

Marine microbial communities play key roles in the marine food webs and in the regulation of many biogeochemical cycles [1, 2]. Marine microplanktonic communities encompass a wide variety of taxonomic and functional groups, like protists, bacteria, or archaea. Phytoplankton and bacterioplankton are the dominant microorganisms in marine ecosystems, and they comprise very dynamic and diverse communities [3, 4]. In the last decades, thanks to the advances in molecular and computational analyses, it became feasible to study the phylogenetic diversity and community composition patterns of marine microorganisms through the sequencing of the 18S rRNA and 16S rRNA genes [5–7]. The combination of the sequence data and the detailed characterization of the environmental conditions represent an excellent approach to explore the influence of the abiotic factors on microbial communities [7].

Earlier studies of bacterioplankton have described their diversity and community seasonality in relation to multiple environmental variables (e.g., solar radiation, temperature, dissolved organic matter (DOM) concentration and quality, or nutrients) [e.g., 4, 8, 9]. For instance, DOM and nutrient supply can vary as a result of water column mixing and stratification, partly driving seasonal patterns in bacterial diversity, composition, and structure [4, 8, 9]. In addition, some experimental studies have demonstrated the influence of compounds released by small-sized phytoplankton through exudation or cell breakage (dissolved organic carbon from phytoplankton, DOC.p) on bacterial taxonomic composition. As an example, the dominance of small phytoflagellates or diatoms promoted shifts in bacterioplankton composition during microcosms experiments [e.g., 10, 11]. DOC.p represents a primary source of organic matter for the bacterial metabolism [12], and about half of the phytoplanktonic organic matter is consumed and remineralized by bacteria [13, 14].

Recent studies suggest that apart from the control exerted by temporal and spatial fluctuations of abiotic variables, biotic factors—in particular the interactions between bacteria and small-sized phytoplankton—are relevant to understand the dynamics of marine microplankton communities [4, 15, 16]. A popular approach to explore the potential role of biotic interactions is the analysis of microbial associations based on correlations [17]. Association analysis is based on co-occurrence or co-exclusion patterns among different taxa. Co-occurrence patterns, which imply coexistence, may result from either positive or negative interactions, such as parasitism (where one-part benefits and the other is negatively impacted), mutualism (where both parts benefit), commensalism (where one organism benefits and the other is neither positively nor negatively influenced) or predation (one organism feeds from other) [18, 19]. By contrast, co-exclusion patterns could reflect allelopathy (secretion of antimicrobial substances) or competitive exclusion among taxa [18, 19]. Many positive or negative microalgae-bacteria interactions involve the exchange of metabolites. For example, the dinoflagellate *Prorocentrum cordatum* provides organic carbon and B_3_ vitamin to the bacteria *Dinoroseobacter shibae*, and in return the bacteria provide B_1_ and B_12_ to the dinoflagellate [20], both partners benefiting from the trade. By contrast, *Alteromonas* sp*.* release chitinase or β-glucosidase that specifically attacks the cell-wall of *Alexandrium tamarense* [21].

Shelf waters off the Ría de Vigo are seasonally affected by upwelling pulses which sustain high levels of productivity [22, 23]. A recent work in the area using DNA-fingerprinting suggests that seasonality in abiotic conditions plays a major role as structuring factor of bacterioplankton communities [9]. The phytoplankton community of the Ría de Vigo is composed mainly by large diatoms (e.g., *Thalassiosira rotula*) in early spring, small diatoms (e.g., *Pseudo-nitzschia* spp. or small *Chaetoceros* spp.), and small flagellates (e.g., *Ostreococcus* spp.) in late spring and summer, and dinoflagellates (e.g., *Tripos furca*) in autumn [23, 24]. Over the winter, phytoplankton abundance is lower compared with the other seasons and benthic species are relatively abundant in the plankton (e.g., *Navicula* spp.) [23]. Yet, a recent study in the area, based on 18S rDNA tag sequencing, revealed that the seasonal succession of small eukaryote operational taxonomic units (OTUs), which were mostly dominated by phytoplankton taxa, seems to be only moderately explained by the environment [24]. To further explore the role biotic interactions may have on microbial dynamics in this productive region, we used the same sample set as in Hernández-Ruiz et al. [24] to simultaneously analyze monthly variations in bacterial community composition (partial 16S rDNA gene sequencing) and function (bacterial biomass, production, and respiration) over 2 years in shelf waters off the Ría de Vigo. The specific objectives of this investigation were as follows: (1) to simultaneously describe, for the first time in this area, spatial and temporal patterns in the bacterioplankton community function and taxonomic composition; (2) to identify which environmental variables explain the variability of the bacterioplankton community composition (BCC); (3) to explore the correlation between bacterial and small-sized eukaryotic community composition (ECC); and (4) to detect co-occurring, and thus potentially interacting, pairs of small phytoplankton and bacteria species, using correlation network analysis. We hypothesize that BCC is coupled with ECC, and that small-sized phytoplankton-bacteria interactions play a significant role in the microbial communities of this productive ecosystem. Based on previous studies, we expect an important influence of environmental factors on temporal and seasonal changes in BCC [4, 8, 9] and a predominance of positive over negative connections between small-sized phytoplankton and bacteria [25, 26].

## Methods

### Sampling

Seawater sampling was carried out monthly from January 2014 to December 2015 in a shelf station off the Ría de Vigo (Spain), three sampling months were missing because of ship technical issues (July and August 2014) or rough weather conditions (December 2015). Seawater was collected from two different depths, near surface (ca. 1 m) and approximately the base of photic zone (30 m). The annual average percentage of photosynthetically active radiation (% PAR) at 30 m in this sampling site is 3.3 ± 1.4%, as estimated in a previous study by Teira et al. [27]. Samples were collected with 5 L Niskin bottles on board R/V José Navaz.

### Environmental Variables

Environmental conditions during the sampling period were previously described in detail by Hernández-Ruiz et al. [24]. Briefly, temperature and salinity were obtained with SBE-25 CTD equipped with Seapoint in situ fluorometer, upwelling index was estimated by calculating the offshore Ekman transport from coastal winds, and precipitation and solar irradiation data were obtained from the Regional Weather Forecast Agency-Meteogalicia (http://www.meteogalicia.gal). Inorganic nutrients were analyzed by standard colorimetric methods with a flow analyzer [28] and size-fractionated chlorophyll-*a* (Chla) concentration was determined from acetone extracts of plankton and measured by the fluorometric method [29]. Dissolved organic carbon (DOC) and total dissolved nitrogen (TDN) were measured in a Shimadzu TOC-V analyzer following the method of Álvarez-Salgado and Miller [30]. Dissolved organic matter fluorescence (FDOM) was measured, following the work of Nieto-Cid et al. [31], at two fixed excitation/emission wavelengths: 320 nm/410 nm (peak M), characteristic of marine humic-like substances, and 280 nm/350 nm (peak T), characteristic of protein-like materials.

### Microbial Metabolic Activity

Prokaryote and eukaryote function-related variables were previously described in detail by Hernández-Ruiz et al. [24]. In brief, prokaryote biomass (PB) was measured using a Becton Dickinson FACSCalibur flow cytometer equipped with a laser emitting at 488 nm [32] and prokaryotic cells were stained with SybrGreen DNA fluorochrome and identified on the basis on their fluorescence and light side scatter (SSC) signature. Biovolume was determined following the empirical calibration described by Calvo-Díaz and Morán [33] and converted into biomass using the allometric relationship from Norland [34]. Heterotrophic prokaryote production (HPP) was estimated by [^3^H]-leucine incorporation method [35], modified as described Smith and Azam [36]. A theoretical leucine to carbon conversion factor of 3.1 kg C mol Leu^−1^ was used [37]. On the other hand, size-fractionated community respiration was calculated using the INT ((iodo-phenyl)–3-(nitrophenyl)–5-(phenyl) tetrazolium chloride) reduction rate method, as described Martínez-García et al. [38] and as described in Hernández-Ruiz et al*.* [24], and the prokaryote respiration (PR) was defined as pico-sized community respiration [24]. Primary production (PP) was estimated as described in detail by Hernández-Ruiz et al*.* [24] and PP rates were calculated using the method described by Marañón et al. [39]. In brief, seawater samples were incubated with 10 μCi of NaH^14^CO_3_ and each incubated sample was measured on a Wallac *β*-scintillation counter [24]. Finally, prokaryotic growth efficiency (PGE) is the amount of new prokaryotic biomass produced per unit of organic C substrate assimilated and was calculated as: PGE = (HPP)/(HPP + PR).

### Microbial Community Composition

Approximately 2–3 L of water samples were sequentially filtered through 20 and 3 μm pore size polycarbonate filters and 0.2 μm pore size Sterivex Filter Units, and immediately frozen in liquid nitrogen and stored at − 80 °C until DNA extraction. DNA retained in 20 μm and 3 μm filters represented microeukaryotes and nanoeukaryotes, respectively. Picoeukaryotes and prokaryotes were collected in the 0.2 μm pore size Sterivex filters. Changes in the ECC during the period of study were assessed by sequencing the V4 region of the 18S rRNA gene and is described in Hernández-Ruiz et al*.* [24]. Nonetheless, in order to compare temporal and spatial changes in bacterial and eukaryotic community composition, eukaryote sequence raw data were reanalyzed for this study in order to incorporate corrections to account for compositional effects (i.e., centered log ratio transformation, clr), not considered in Hernández-Ruiz et al*.* [24].

In all, 42 DNA samples were amplified for partial 16S rRNA gene sequencing. DNA from pico-sized plankton (< 3 μm diameter) was extracted using PowerSoil ® DNA isolation Kit (MoBio Laboratories Inc., CA, USA) according to the manufacturer’s instructions. The DNA concentration was quantified using a Qubit® 2.0 fluorometer and Qubit dsDNA HS Assay Kit (Thermo Fischer Scientific Inc, Massachusetts, USA). The extracted DNA was amplified using the primers 515F-Y (5′-GTGYCAGCMGCCGCGGTAA-3′) and 926R (5′-CCGYCAATTYMTTTRAGTTT-3′). These primers target the V4-V5 hypervariable regions of the 16S rRNA gene [40]. Amplified regions were sequenced with Illumina MiSeq platform (paired-end reads; 2 × 300 bp). Sequence reads were analyzed as described in Logares [41]. In short, raw reads were corrected by Bayes Hammer [42] following the Schirmer et al. [43] method. Subsequently, paired-end reads were merged with PEAR [44] and the longer sequences (> 200 bp) were quality-checked and dereplicated using USEARCH [45]. OTU abundances were acquired by mapping back reads to OTUs at 99% similarity. BLAST [46] was used for taxonomic assignment of 16S OTUs, against SILVA 123 database. OTUs assigned to chloroplasts, mitochondria, or eukaryotes were removed. As archaea were poorly represented in our sample set, archaeal OTUs were also excluded for this study. Finally, after the computing analysis we subsampled the OTU table to the lowest number of reads, which was 5274. The subsampling was carried out with the “vegan” R-package. The sequence abundances of the subsampled OTU table were transformed by the clr. The clr transformation was performed to address compositionality data and obtain more realistic data fitted within an Euclidean space [47]. Clr transformation was conducted with the “compositions” R-package. Before the clr transformation, zeroes were replaced by the minimum value divided by 2 according to Fernandes et al. [48].

The diversity indices that we calculated in each time point and depth were richness and alpha diversity. Bacterial richness was calculated by the number of distinct OTUs per sample, and alpha diversity was measured by Shannon index, *H*. They were calculated with the “diversity” function in the “vegan” R-package.

### Statistical Analyses

Differences in prokaryote function-related variables between depths and seasons were analyzed by the non-parametric Kruskal–Wallis (K-W) and Mann–Whitney (M-W) tests. We used these tests because PB, HPP, and PGE had non-normal distributions even after transformation. On the other hand, Student *t* test and ANOVA were performed to compare differences between richness and Shannon index among depths, years and seasons. Diversity indices followed a normal distribution. Normal distribution was tested by Shapiro–Wilk test. All of the tests were executed with the “stats” R-package.

All data used for multivariate statistical analyses were previously transformed. The read abundances of eukaryote and bacteria OTUs were transformed using clr transformation. Environmental (solar radiation (Irr), precipitation (Pre), water temperature (Twat), dissolved organic carbon (DOC), total dissolved nitrogen (TDN), dissolved organic matter fluorescence (FDOM), phosphate (PO_4_), ratio dissolved inorganic nitrogen and phosphate (DIN.P), silicate (SIO_2_), and upwelling index (UI)) and functional variables (pico-, nano-, and micro-sized primary production (PP.p, PP.n, PP.m); heterotrophic prokaryote production (HPP); prokaryote biomass (PB); pico-, nano-, and micro-sized chlorophyll-*a* (Chla.p, Chla.n, Chla.m); and pico-, nano-, and micro-sized community respiration (CR.p, CR.n, CR.m)) were normalized using the following equation: *X*_*n*_ = ( *X*_*i*_—$\overline{X }$)/*S*_*x*_. Here, *X*_*i*_ represents the original variable value, $\overline{X }$ represents the mean of the original variable, and ${S}_{x}$ represents the standard deviation of the original variable.

The multivariate redundancy analysis (RDA) was applied to extract and summarize the variation in a set of response variables (in this case eukaryotic and bacterial community composition in the different samples) that can be explained by a set of explanatory environmental variables. PERMANOVA was performed to evaluate significant differences in BCC between different depths and sampling seasons. The significance of the explanatory variables was examined by permutation analysis (permutations = 999) [49]. RDA and statistical tests were performed with the R-package “vegan” and RDA plots were constructed using “ggplot2” and “ggord” R-packages. To evaluate and describe associations between the relative abundance of major bacteria taxa and environmental and functional variables, we constructed a heatmap using Spearman correlations. Heatmap clustering was based on Euclidean distances. Spearman correlations were calculated using “Hmisc” R-package and heatmaps were constructed using “ComplexHeatmap” R-package.

A partial Mantel test was used to study the relationship between the Euclidean distance matrices built from (a) normalized environmental variables, (b) normalized functional variables, (c) clr abundance of small-sized eukaryotes (< 20 μm), and (d) clr abundance of bacteria. Partial Mantel correlation uses partial correlation conditioned on a third matrix, and the Mantel coefficient is algebraically equivalent to the Pearson correlation coefficient (permutations = 999, *p* < 0.05). Partial Mantel tests were computed using “vegan” R-package.

### Network Analysis

In order to explore the potential interactions between bacteria and eukaryotes, we built a co-occurrence network based on Spearman correlation between the 50 most abundant and frequent (present in more than 50% of the samples) bacteria and eukaryote OTUs. We used the habitat filtering (HF) algorithm to correct for the effects that the different environmental conditions of the two sampling depths may have on the microbial correlation network analysis. The HF algorithm was proposed by Brisson et al*.* [50] and essentially corrects the abundance of each OTU in each sample by the mean abundance of that OTU in its habitat. Benjamini and Hochberg correction was applied to control for false positives [51], and the co-occurrence network between eukaryote and bacteria OTUs was built including only correlations with a significance cutoff of *p* < 0.01 [50].

In our study, we focused on the relationships between bacteria in the size fraction < 3 μm and eukaryotes in the size fraction < 20 μm. Since most bacteria < 3 μm are free-living, most of the detected potential connections will not imply intimate interactions such as those occurring in parasitic (except those parasites that present free-living stages) or symbiotic relationships. The small plankton (< 20 μm size fraction) is a significant fraction of the microbial plankton and largely contribute to the total microbial plankton biomass in shelf waters of NW-Spain [52]. Yet, the taxonomic composition of this small plankton fraction was described for the first time in the related study by Hernández-Ruiz et al. [24].

Node degree and neighborhood connectivity were calculated using the network analyzer tool in Cytoscape 3.8.2. The network was visualized with “igraph” and “ggplot2” R-packages.

## Results

### Environmental and Phytoplankton Function-Related Variables

As previously described in Hernández-Ruiz et al. [24], surface temperature was higher (average: 15.2 ± 2.5 °C) than at 30 m temperature (average: 14.4 ± 2.4 °C), with warmer surface waters during late summer and autumn in 2014 than in 2015 [24]. In both years, upwelling conditions dominated from March to August, while downwelling conditions dominated in January and February [24]. Dissolved inorganic nitrogen (DIN) concentration in surface waters was higher in winter than in summer, and higher in 2014 than in 2015 [24]. DOC and DON were higher in surface waters (average DOC: 82 ± 7.8 µmol L^−1^; average DON: 7.9 ± 2.7 µmol L^−1^) than at 30 m depth (average DOC: 69.3 ± 5.3 µmol L^−1^; average DON: 7 ± 2.2 µmol L^−1^) and showed relatively low variation between years [24]. The fluorescence of protein-like DOM (FDOM.T) displayed much higher values in 2015 than in 2014, and in spring and early autumn than in winter and summer in 2015 [24].

PP and Chla were broadly higher in surface waters (average PP: 67.5 ± 112. 3 µg C L^−1^ day^−1^; average Chla: 2.9 ± 3 µg L^−1^) than at 30 m depth (average PP: 13.2 ± 18.7 µg C L^−1^ day^−1^; average Chla: 1.5 ± 2.2 µg L^−1^) [24]. PP and Chla had similar seasonal trends, with higher values in spring and late summer and lower during winter and summer at both depths [24]. Particularly outstanding were the PP and Chla peaks in May 2014 at both depths and in September 2015 in surface waters [24].

### Prokaryote Function-Related Variables

**Fig. 1:**
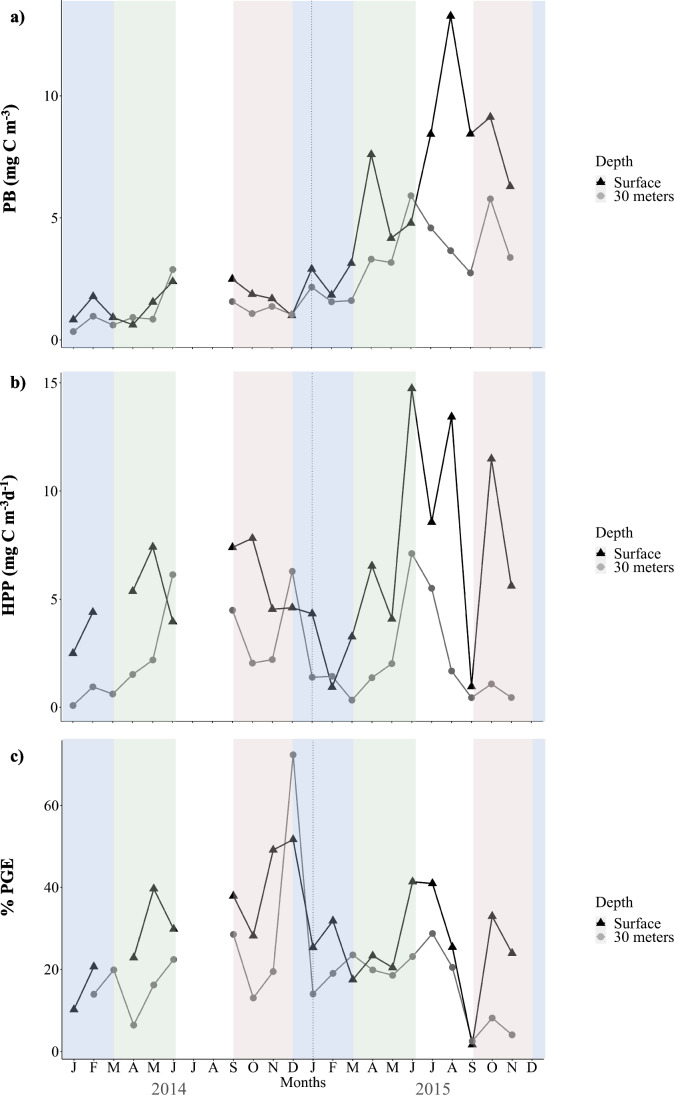
**a** Prokaryote biomass (PB), **b** heterotrophic prokaryote production (HPP), and **c** prokaryotic growth efficiency over January 2014 and December 2015 in surface waters (triangles and black line) and at 30 m depth (circles and grey line). Colored shades represent the seasonal trend in winter (blue), spring (green), summer (white), and autumn (brown)

PB, HPP, and PGE displayed more temporal variability in surface waters than at 30 m depth and registered, in general, fluctuations between seasons and years (Fig. 1).

PB throughout the whole period of study averaged 4.1 ± 3.5 mg C m^−3^ and 2.4 ± 1.6 mg C m^−3^ in surface waters and at 30 m depth, respectively, and 1.3 ± 0.7 mg C m^−3^ and 4.9 ± 2.9 mg C m^−3^ during 2014 and 2015, respectively (Fig. 1a). PB showed significantly higher values in 2015 than in 2014 (M-W test, *n* = 40, *p* < 0.01), but there were not significant differences between surface and 30 m depth (M-W test, *n* = 40, *p* > 0.05). PB displayed significant changes along seasons (K-W test, *n* = 40, *p* < 0.05), with higher values in early spring and summer than in winter and autumn, and a remarkable increase in August 2015 (Fig. 1a). HPP throughout the whole period of study averaged 6.1 ± 3.7 and 2.4 ± 2.2 mg C m^−3^ day^−1^ in surface waters and at 30 m depth, respectively (Fig. 1b). Surface HPP was higher than at 30 m depth (M-W test, *n* = 40, *p* < 0.01). There were not significant differences between HPP during 2014 and 2015 (M-W test, *n* = 40, *p* > 0.05). Interestingly, there was a strong increase in HPP in June–August 2015, particularly relevant in surface waters, followed by a sharp decrease in September 2015 (Fig. 1b). PGE throughout the whole study period averaged 28.7 ± 12.4% and 19.7 ± 14.4% in surface waters and at 30 m depth, respectively (Fig. 1c). PGE was significantly higher in surface waters than at 30 m depth (M-W test, *n* = 40, *p* < 0.05) and did not differ between 2014 and 2015 (M-W test, *n* = 40, *p* > 0.05). PGE registered the highest values in December 2014, and the lowest values in September 2015, at both depths (Fig. 1c).

### Bacterial Diversity and Composition

**Fig. 2:**
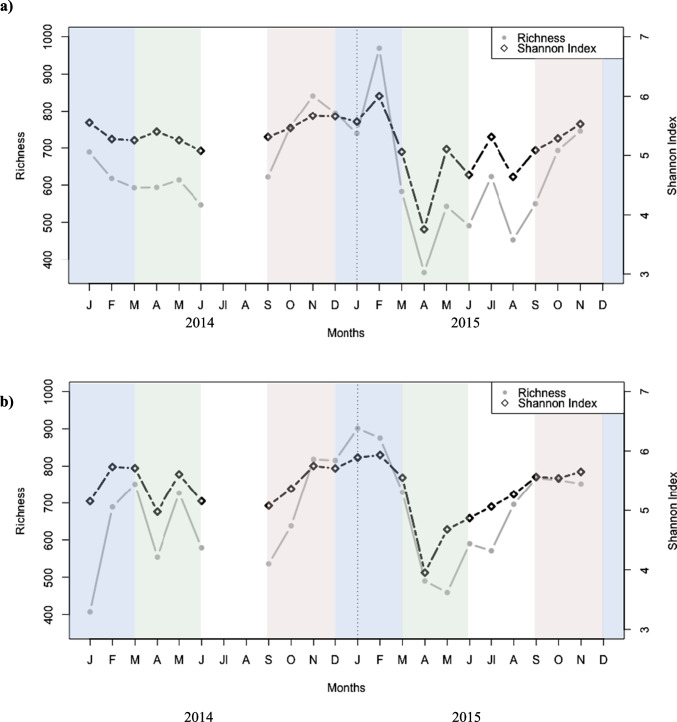
Shannon index and richness from bacteria over 2014 and 2015 in surface waters (**a**) and at 30 m depth (**b**). Colored shades represent the seasonal trend in winter (blue), spring (green), summer (white), and autumn (brown)

Richness and Shannon index (*H*) did not differ between depths (richness: *t*-test, *n* = 42, *p* > 0.05; Shannon index: *t*-test, *n* = 42, *p* > 0.05), and both indices were significantly different among seasons, with higher values in winter and autumn than in spring and summer (richness: ANOVA, *n* = 42, *p* < 0.0001; *H*: ANOVA, *n* = 42, *p* < 10^−5^) (Fig. 2a and b). At both depths, OTU richness and* H* were lower in 2015 than in 2014 and displayed a sharp minimum of both values in April 2015 (Fig. 2a and b). Bacterial richness averaged 711.9 ± 155.1, 761.6 ± 61.3, 546 ± 90.2, and 602.2 ± 97.9 in winter, autumn, spring, and summer, respectively, while alpha-diversity (measured by *H*) averaged 5.6 ± 0.3, 5.6 ± 0.2, 4.9 ± 0.5, and 5.2 ± 0.3 in winter, autumn, spring, and summer, respectively (Table S1).

**Fig. 3:**
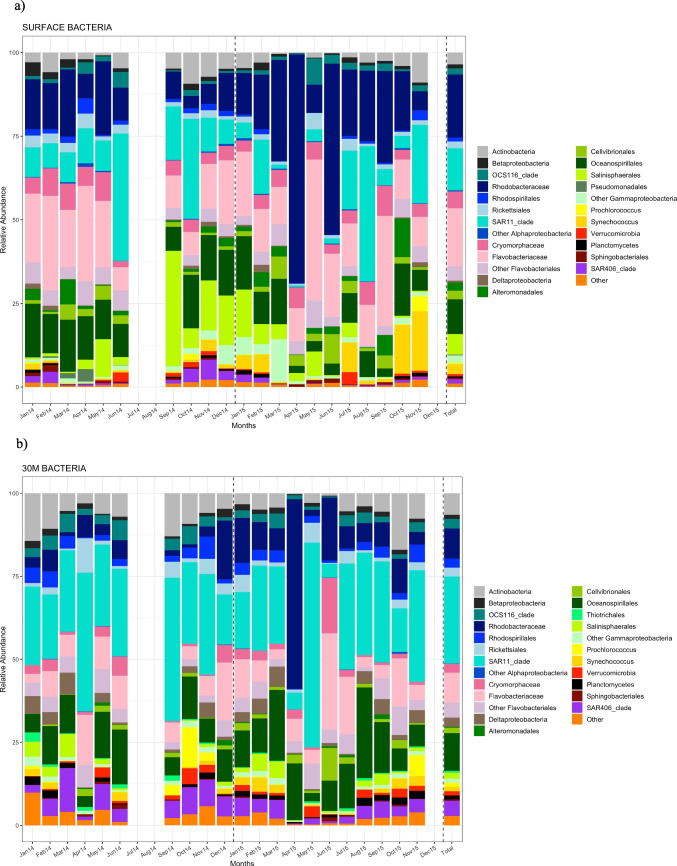
**a** Temporal variation in the relative contribution of reads to the major taxonomic groups of bacteria in surface waters over 2024 and 2015. The last bar represents averaged composition over the sampled period. **b** Temporal variation in the relative contribution of reads to the major taxonomic groups of bacteria at 30 m depth over 2024 and 2015. The last bar represents averaged composition over the sampled period

The BCC showed temporal variability and differed between depths (Fig. 3a and b). Approximately 80% of the sequences were affiliated with *Alphaproteobacteria*, *Flavobacteria*, and *Gammaproteobacteria* at both depths. The average percentage of these classes in surface waters was ~ 37% for *Alphaproteobacteria,* ~ 27% for *Flavobacteria* and ~ 24% for *Gammaproteobacteria*. In surface waters, *Rhodobacteraceae* were the dominant family (relative abundance: ~ 19%), followed by *Flavobacteriaceae* (relative abundance: ~ 17%), and SAR11 clade (relative abundance: ~ 13%) (Fig. 3a). *Rhodobacteraceae* were more abundant in summer and spring and in 2015 compared to 2014. In April 2015, most *Rhodobacteraceae* belonged to the genus *Amylibacter* sp. (~ 83% of *Rhodobacteraceae* reads). The relative abundance of SAR11 increased during summer and decreased during winter in both years and they were relatively more abundant in spring 2014 than in spring 2015. *Cyanobacteria*, *Gammaproteobacteria* and SAR406 became more abundant across autumn and winter in surface waters (Fig. 3a).

At 30 m depth, the dominant bacteria class was *Alphaproteobacteria* (relative abundance: ~ 44%), dominated by the SAR11 clade (relative abundance: ~ 26%) and *Rhodobacteraceae* (relative abundance: ~ 9%), followed by *Gammaproteobacteria* (relative abundance: ~ 17%) and *Flavobacteria* (relative abundance: ~ 16%) (Fig. 3b). The SAR11 clade was more abundant at 30 m depth than in surface waters and its relative abundance was quite constant throughout the period of study except for a sharp decrease in April 2015 (relative abundance: ~ 5%) and a subsequent increase in May 2015 (relative abundance: ~ 62%). *Rhodobacteraceae* were less abundant at 30 m depth than in surface waters and their relative abundance increased in 2015 compared to 2014, being the dominant group in April 2015 (relative abundance of ~ 69% in surface waters and ~ 57% at 30 m depth). As in surface waters, at 30 m depth most *Rhodobacteraceae* in April 2015 belonged to *Amylibacter* sp. (~ 90% of *Rhodobacteraceae* reads). *Salinisphaerales*, *Rhodobacteraceae*, and *Flavobacteriaceae* were less abundant at 30 m depth than in surface waters (Fig. 3b).

### Spatial and Temporal Patterns in Microbial Community Composition in Relation to Environmental and Functional Variables

**Fig. 4:**
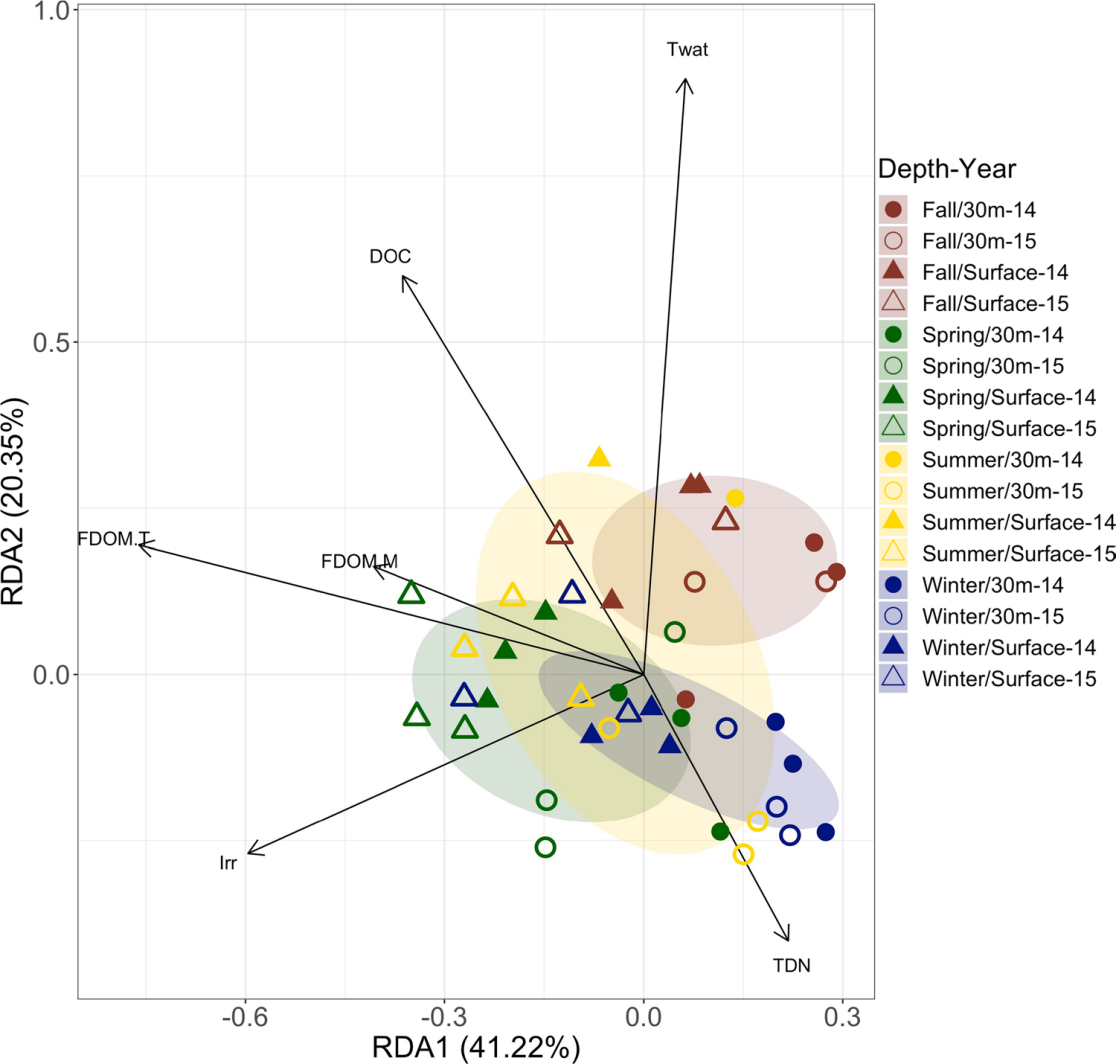
Redundancy analysis (RDA) of bacterial community. Filled and open symbols represent samples from 2014 and 2015, respectively. Circles represent samples from 30 m and triangles from surface. Colored ellipses highlight the divergence of the samples in summer (yellow), fall (brown), winter (blue), and spring (green). The arrows represent the significant variables that explained variability in the structure of the community. Abbreviations: Twat (temperature of water), DOC (dissolved organic carbon), FDOM.M (humic-like dissolved organic matter fluorescence), FDOM.T (protein-like dissolved organic matter fluorescence), Irr (solar radiation), and TDN (dissolved total nitrogen)

RDA was performed to study the patterns of temporal (intra- and interannual) and spatial (depth) variability of BCC. RDA revealed that BCC was significantly influenced by depth (PERMANOVA, *n* = 42, *p* = 0.001) and by the sampling season (PERMANOVA, *n* = 42, *p* = 0.001). The first two canonical axes jointly explained 61.6% of the total variance of BCC, with the first axis alone explaining 41.2%. The significant variables that explained the variability in this model were: Twat, DOC, FDOM.M, FDOM.T, Irr, and TDN. The RDA plot showed that changes in TDN and Twat mostly explained differences in BCC between autumn and winter samples. On the other hand, DOC, FDOM.M, FDOM.T, and Irr partially explained differences in BCC between surface and 30 m depth samples (Fig. 4). For the sake of comparison, we repeated the analysis made in Hernández-Ruiz et al*.* [24] but using the clr transformation applied in the present work (Fig. S1) and we found that 48.6% of the variability in the ECC of the small size-fraction (0.2–20 μm cell-size) was explained by environmental variables (Fig. S1). The RDA performed using clr-transformed ECC data showed two clear communities, one in spring–summer which was explained by the Irr, UI, and Chla, and another one in autumn–winter related to DOC, Chla.p, Pre, and FDOM.M (Fig. S1).

**Fig. 5:**
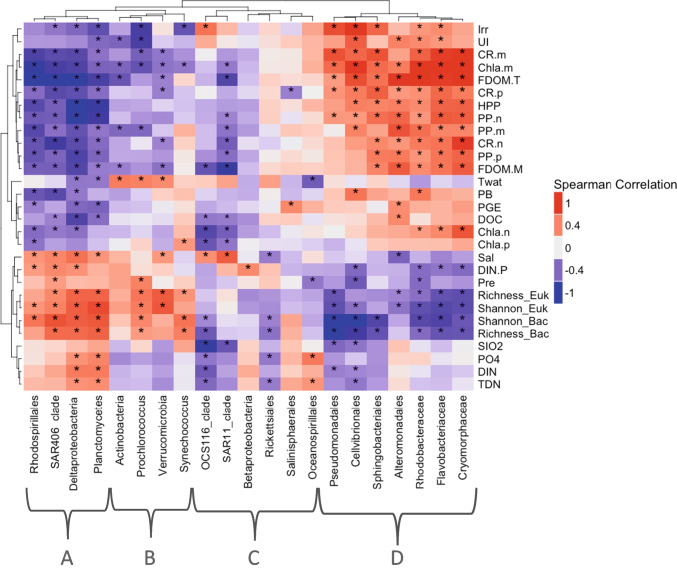
Spearman correlation of bacteria with environmental and functional variables: salinity (Sal); solar radiation (Irr); temperature of water (Twat); upwelling index (UI); dissolved inorganic nitrogen and phosphate ratio (DIN.P); precipitation (Pre); eukaryote and bacteria Shannon index (Shannon_euk and Shannon_bac); phosphate (PO_4_); humic-like dissolved organic matter fluorescence (FDOM.M); protein-like dissolved organic matter fluorescence (FDOM.T); silicate (SIO_2_); total dissolved nitrogen (TDN); dissolved inorganic nitrogen (DIN); pico-, nano-, and micro-sized chlorophyll-*a* (Chla.p, Chla.n, and Chla.m); pico-, nano-, and micro-sized community respiration (CR.p, CR.n, and CR.m); prokaryote biomass (PB); heterotrophic prokaryote production (HPP); prokaryotic growth efficiency (PGE); pico-, nano-, and micro-sized primary production (PP.p, PP.n, and PP.m) and dissolved organic carbon (DOC). Dendrograms represent clustering of bacteria based on their correlations with abiotic and biotic variables (Euclidean distance). Asterisks symbolism the significant correlations (*p* < 0.05)

Four different clusters of bacterial taxa (A, B, C, and D) were obtained based on their correlation with environmental and functional variables (Fig. 5). Overall, cluster A (including SAR406 and *Deltaproteobacteria*, among others) showed negative correlations with Irr, UI, CR, Chla.m, HPP, PP, DOC, and DOM (*n* = 42, *p* < 0.05). Furthermore, cluster A showed strong positive correlations with salinity (Sal), and richness and *H* of bacteria and small-sized eukaryotes (*n* = 42, *p* < 0.05) (Fig. 5). Conversely, cluster D (including *Rhodobacteraceae* and *Cryomorphaceae*, among others) showed strong positive correlations with Irr, UI, CR, Chla*.*m, HPP, PP and FDOM.T and FDOM.M (*n* = 42, *p* < 0.05). This cluster was negatively correlated with DIN.P, richness and H of bacteria and small-sized eukaryotes (*n* = 42, *p* < 0.05) (Fig. 5). On the other hand, cluster B (including *Prochlorococcus* and *Synechococcus*, among others) and C (including SAR11, and *Oceanospirillales* among others) showed, in general, lower number of correlations than the cluster A and D. Overall, cluster B showed negative correlations with Chla.m and positive correlations with Twat, richness and* H* of bacteria and small-sized eukaryotes (Fig. 5). Cluster C showed weak correlations with environmental variables, with the exception of OCS116 and SAR11 clades, which showed strong negative correlations with most variables and positive correlations with Sal and, in the case of OCS116 with Irr too (Fig. 5).

Spearman correlations of the different eukaryotic taxa (small size-fraction, 0.2–20 μm cell-size) studied with environmental and functional variables were weaker than those of bacteria (Fig. S2). Within phytoplankton taxa, *Cryptophyceae* was the group showing more significant correlations with environmental and functional variables (Fig. S2). By contrast, *Bacillariophyceae* only showed two positive correlations with PO_4_ and UI (Fig. S2).

### Links Between Bacterial Community, Eukaryotic Community, Environmental Factors, and Functional Variables

**Fig. 6:**
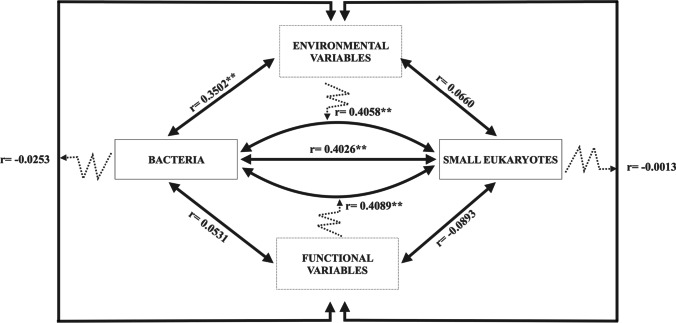
Mantel analysis between the distance matrices of clr abundance of bacteria, clr abundance of small size (0.2–20 μm size fraction) eukaryotes, environmental variables (solar radiation, precipitation, upwelling index, temperature of water, dissolved organic carbon, total dissolved nitrogen, humic-like dissolved organic matter fluorescence, dissolved inorganic nitrogen and phosphate ratio, protein-like dissolved organic matter fluorescence, phosphate and silicate), and functional variables (pico-, nano-, and micro-sized chlorophyll-*a*; pico-, nano-, and micro-sized community respiration; prokaryote biomass; prokaryote production; pico-, nano-, and micro-sized primary production). Numbers adjacent to arrows are Mantel statistic r and dotted arrows represent the effect of the third distance matrix on the relationship. Significance levels are as follows: * *p* ≤ 0.05 and ** *p* ≤ 0.01

The Mantel test revealed a significant correlation between ECC of the small size fraction (0.2–20 μm cell-size) and BCC (*n* = 38, *r* = 0.4, *p* < 0.01), as well as a significant partial correlation between ECC of the small size fraction (0.2–20 μm cell size) and BCC after controlling for the effects of environmental factors (*n* = 38, *r* = 0.41, *p* < 0.01) or the effects of functional variables (*n* = 38, *r* = 0.41, *p* < 0.01) (Fig. 6). BCC had a significant correlation with environmental factors (*n* = 38, *r* = 0.35, *p* < 0.01); but not with functional variables (*n* = 38, *r* = 0.05, *p* > 0.05). By contrast, ECC of the small size fraction (0.2–20 μm cell size) did not have a significant correlation with the functional (*n* = 38, *r* =  − 0.09, *p* > 0.05) nor with the environmental variables (*n* = 38, *r* = 0.07, *p* > 0.05). On the other hand, the environmental and functional variables did not show a significant relationship between them (*n* = 38, *p* > 0.05) (Fig. 6).

### Co-occurrence Networks Between Bacteria and Eukaryotes

**Fig. 7:**
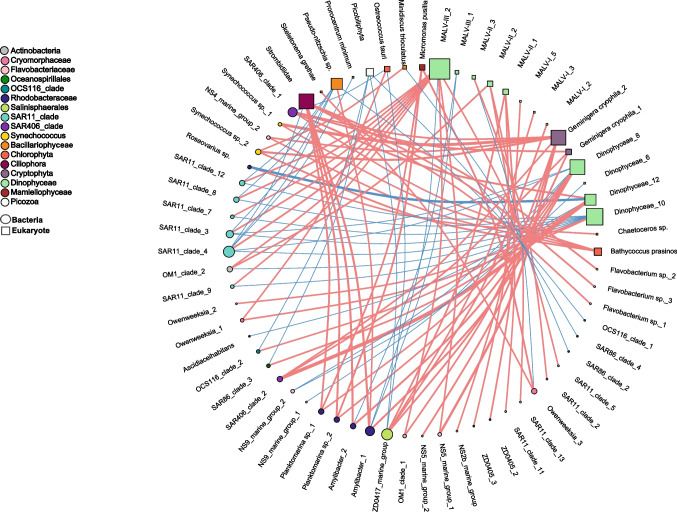
Small size (0.2–20 μm size fraction) eukaryotes and free-living bacteria (< 3 µm size fraction) co-occurrence network with habitat filtering correction. Edge color refers to the type of relationship with significant connection between bacterial and eukaryotic OTUs, red for positive and blue for negative. The size of the nodes is proportional to node degree and the width of edges is proportional to Spearman correlation coefficient. Square and circular nodes represent eukaryotes and bacteria, respectively. Bacterial nodes are colored according to color scheme in Fig. 3

It is important to note that only eukaryote-bacteria connections were considered in this analysis. Small size fraction (0.2–20 μm cell size) eukaryote and free-living bacteria (< 3 µm size fraction) network with HF correction included a total of 92 significant relationships (*n* = 41, *p* < 0.01), of which 60 were positive and 32 negative (Fig. 7). Eukaryote OTUs had, on average, more connections with bacteria (3.8 ± 3.0) than bacterial OTUs with eukaryotes (2.2 ± 1.4) (Table S2 and Table S3) and, conversely, averaged neighborhood connectivity, which is the average number of neighbors of the connected nodes, was higher for bacteria (6.1 ± 2.6) than for eukaryotes (3.2 ± 1.4) (Table S2 and Table S3). The most connected eukaryotic node was MALV-III_2 (*Dinophyceae*) (degree = 11), followed by *Dinophyceae*_10 (degree = 9), and the most connected bacterial nodes were ZD0417_marine_group (*Salinisphaerales*) (degree = 6) and SAR11_clade_4 (degree = 6). The network showed that most potential interactions occurred between *Dinophyceae*, and *Rhodobacteraceae* or SAR11_clade (Fig. 7). Moreover, *Dinophyceae*, MALV-II and MALV-III groups showed many different negative interactions with bacteria, particularly with *Amylibacter* sp. (*Rhodobacteraceae*), SAR11, and NS9_marine_group (*Flavobacteria*). *Crypthophyta* showed strong and positive associations with *Synechococcus*, SAR406 and *Flavobacteria*. *Chlorophyta* showed repeated positive associations with *Flavobacteria* and *Synechococcus*. Within the *Bacillariophyceae*, *Pseudo-nitzschia* sp*.* presented many negative correlations with SAR11 and OC116 (*Alphaproteobacteria*), and showed only one positive correlation with *Amylibacter* sp*.* (*Rhodobacteraceae*) (Fig. 7). By contrast, *Strombidiidae* (*Ciliophora*) showed strong and positive correlations with *Rhodobacteraceae* and *Flavobacterium*. The strongest positive correlations observed occurred between *Dinophyceae*_12 and SAR406_clade_2 and between *Geminigera cryophila*_2 (*Cryptophyta*) and *Synechococcus* sp._1 (*Cyanobacteria*), while the strongest negative correlation was found between *Dinophyceae*_12 and *Roseovarius* (*Rhodobacteraceae*) (Fig. 7).

## Discussion

This study provides the first detailed description of bacterial community composition and prokaryote function-related variables (biomass, production, and growth efficiency) over 2 years in shelf waters in the upwelling system off the Ría de Vigo (NW-Spain). Our results reveal a close connection between bacterial community composition and environmental factors, and the key role of bacteria as a structuring factor of the eukaryotic community, mostly driven by positive connections between phytoplankton and bacteria.

### Temporal and Vertical Patterns in Bacterial Diversity and Composition

*Alphaproteobacteria*, dominated by SAR11 clade and *Rhodobacteraceae* family, *Gammaprotebacteria*, and *Flavobacteriia* (*Bacteroidetes*), were the most abundant classes at both depths, in agreement with these bacterial taxa being widely distributed in marine epipelagic waters [7, 53, 54].

Our results suggest a certain degree of seasonality in the diversity of marine bacterial communities in this ecosystem, in accordance with previous studies in temperate and (sub)-tropical areas [e.g., 54–56]. The higher diversity of bacterial communities in autumn–winter compared to other months was previously described by Hernando-Morales et al. [9] in the area and is probably related with the lower availability of resources (e.g., phytoplankton-derived DOM) in winter compared to the rest of the seasonal cycle. The upwelling conditions in summer, provide nutrients that can be quickly utilized by some phytoplankton species which may bloom and contribute to the pool of bioavailable substrates for bacterial growth [e.g., 9, 57]. The increased availability of DOM may trigger the bloom of some bacterial populations which may become dominant, and, consequently, decrease the diversity of the community. As an example, the sharp decrease in richness and *H* of the bacterial community observed in April 2015 coincided with a bloom of *Amylibacter* sp.

BCC showed vertical and seasonal variability probably related with changes in water temperature, solar radiation or resources (i.e., DOM), in agreement with previous time-series studies in the area [9] and in other regions like in San Pedro Channel off the coast of Southern California [54], in the Western English Channel located off the southern coast of the UK [55] and in Bermuda Atlantic time-series(BATS) station in the west of the Atlantic Ocean [58]. BCC similarities between both depths in winter and autumn samples are probably related with the homogenization of the communities due to water column vertical mixing [9, 54]. Moreover, our results strongly suggest that BCC variability is mostly modulated by the quantity and quality of the available DOM (i.e., by DOC, TDN, FDOM.T, and FDOM.M), as proposed in previous investigations [e.g., 11, 59, 60].

The dominance of SAR11 clade in stratified summer waters, was likely associated to low nutrient availability [24], in line with SAR11 being adapted to oligotrophic conditions [53, 54]. This is coherent with the negative correlation of this group with PP. Similarly, the generalized absence of *Synechococcus* (*Cyanobacteria*) during upwelling months (i.e., March to September; [24]) is in accordance with previous studies in Monterey Bay, California, where *Synechococcus* were negatively correlated with chlorophyll levels during upwelling period [61, 62]. By contrast, the sporadic presence of *Prochlorococcus* (*Cyanobacteria*) in autumn, coinciding with the transition period between upwelling and downwelling conditions [24] suggests that ocean currents introduced this typically oceanic taxa [63] into shelf waters, as also observed in the southern Bay of Biscay [33].

The significantly positive correlations between the relative abundances of the groups of the cluster D and phytoplankton-related variables (e.g., PP or Chla.n and Chla.m) suggest a link between these bacterial taxa and phytoplankton bloom dynamics in this productive temperate ecosystem. These results are coherent with the conception of bacterial species succession being linked to the availability of DOM derived from primary producers [e.g., 16, 64]. On the other hand, the negative correlations between *Rhodospirillales, Deltaproteobacteria*, SAR406 and *Planctomycetes* (cluster A) with phytoplankton related-variables agrees with their preference for autumn–winter conditions (particularly at 30 m depth).

### Links Between Bacterial and the Eukaryote Communities

While BCC in this temperate ecosystem appeared to be significantly related with environmental variables, ECC of the small size fraction (0.2–20 μm cell size) was not significantly explained by environmental variables. This result suggests that environmental factors may be more important for structuring the bacteria than the eukaryotic community. Moreover, multivariate analyses consistently showed that the composition of the small-sized eukaryotic community (dominated by phytoplankton taxa) was better predicted from the composition of the associated bacterial communities than from environmental contextual variables, as previously reported in the study area [65] and elsewhere [e.g., 66, 67]. This result is coherent with the notion that biotic interactions may play a more critical role than previously assumed as microbial community structuring factors [e.g., 15, 25, 26]. It could be thus hypothesized that the seasonal succession patterns described by Hernández-Ruiz et al. [24] for the small eukaryotes in this region could be mostly driven by seasonal changes in the BCC.

In aquatic ecosystems, eukaryote-bacterioplankton positive connections are expected to dominate over negative connections, and typically involve the exchange of extracellular molecules like vitamins, hormones, sugars, or amino acids [15, 20, 21]. Our network showed mostly positive connections between bacteria and small eukaryotes, as also observed with similar approaches by Lima-Méndez et al. [25] or Pacheco and Segrè [26]. Nevertheless, the predominance of the positive connections over the negative ones could arise because negative associations are more difficult to detect from the observational data as they may imply that one of the interacting pairs is excluded or in very low abundance, while positive correlations are easier to find among the abundant and frequent taxa selected for the analysis because both taxa will be present in the samples [68]. Eukaryotes displayed more links (edges) with bacteria than bacteria with eukaryotes in our network, which might indicate that small-sized eukaryotes may need many different bacteria to fulfil growth requirements [69].

The built network revealed interesting connections between microbial groups in this productive ecosystem, such as those suggesting mutualistic relationships between *Dinophyceae* and *Rhodobacteraceae*. Previous works have described that auxotrophic diatoms and dinoflagellate, requiring B_12_, B_1_, and/or B_7_ to grow, can obtain these compounds from bacteria belonging to the *Rhodobacteraceae* family [20, 70–72]. Dinophyceae may provide organic carbon and/or vitamins (i.e., B_3_) to *Rhodobacteraceae*, and, in return, *Rhodobacteraceae* would supply B-vitamins (B_1_ or B_12_) to *Dinophyceae* [20, 73, 74]. The significant co-occurrence between *Pseudo-nitzschia* sp. and *Chaetoceros* sp. with *Rhodobacteraceae* (*Amylibacter* sp. and *Roseovarius* sp., respectively) could also represent mutualism or commensalism involving exchange of B vitamins, as both diatoms are B vitamin auxotrophs [71, 72, 75]. A previous study [76] also found specific interactions between diatoms and bacteria during spring a summer blooms in the Southern Ocean. Another example of potential mutualism/commensalism in our network would be between *Chlorophyta* and *Cryptophyta* with *Synechococcus*. In this case, this potential interaction could be mediated by the supply of pseudocobalamin (a chemical variant of B_12_) by *Synechococcus*, which could be remodeled by *Chlorophyt*a and *Cryptophyta* to obtain cobalamin [77, 78]. The strong positive correlation found between SAR406 and *Dinophyceae* could be related to the recently described heme auxotrophy of this not-yet cultured bacteria [79]. Interestingly, SAR11 showed many positive relationships with *Dinophyceae* and MALV groups. SAR11 bacteria have an unusual requirement for a wide range of substrates for growth as a result of their reduced genomes, which might be met by establishing complex interactions with other eukaryotes [53, 80, 81]. *Flavobacteriia*, in general, showed positive connections with *Dinophyceae*, *Strombidiidae* (*Ciliophora*), *Geminigera cryophila* (*Cryptophyta*), and *Chlorophyta*, which may reflect either metabolic exchange or predation. Even though predation has been commonly associated with co-exclusion patterns [82], predator–prey dynamics may also result in positive correlations, particularly when both predator and prey are rare and the predator effectively tracks the prey, resulting in significant co-occurrence patterns (see review by Thurman et al. [83]).

Our results also suggest potential negative relationships between eukaryotic and prokaryotic microplankton in this system, which might involve competition, between bacteria and eukaryotes for limiting nutrients, predation or antagonistic interactions, mediated by the release of bactericides or algaecides [15, 18, 19, 84]. The strongest negative connection observed in this ecosystem over the period of study occurred between *Dinophyceae* and *Roseovarius* sp., which might be an example of bacterial algicidal activity. Some species of this bacterial genus can produce several algicides, like N-9-hexadecenoylalanine methyl (build from a fatty acid and an amino acid) isolated from *Roseovarius lutimaris*, that inhibit the growth of diatoms [84]. On the other hand, there was an important number of negative connections between *Pseudo-nitzschia* sp. and SAR11 and OCS116 could be related with the ability of *Pseudo-nitzschia* cells to produce domoic acid and inhibit the growth of specific bacteria [85–87]. Lastly, the negative connections could also result from predator–prey relationships, as many predator–prey dynamics result in negative abundance correlations, due to time-delays between both populations. In marine planktonic systems, protists are a major source of mortality for both heterotrophic and autotrophic bacteria [88]. In the case of the negative connections with *Flavobacteriia*, the fact that *Ciliophora*, *Cryptophyta*, and *Dinophyceae* have been previously shown as heterotrophic or mixotrophic groups that may behave as bacterivores [e.g., 66, 89, 90], would support the hypothesis of predation. In this study many negative connections were found between SAR11 and *Dynophyceae*, SAR86 and *Picobiliphyta*, or *Amilybacter* sp. and MALV. Several experimental studies suggest that the prey size and the physiological state could be important selective factors for bacterivores in the water column [91, 92]. Even though *Picobiliphyta* have very small cell size (< 5 μm) and could preferentially prey on small-sized bacteria, bacterivory in these small protists has not been demonstrated so far [93]. MALV have been described as parasites with an ephemeral free-living stage, however, to the best of our knowledge, bacterivory has not been described within this group. Therefore, most of the aforementioned negative connections are likely reflecting either competitive or antagonist interactions.

Taken together, correlation and co-occurrence analyses revealed a strong and significant connection between free-living bacteria and small-sized eukaryotes, which provided support for the potential role of biotic interactions as community structuring forces even beyond the phycosphere scale. The abundant associations among bacteria and small-sized eukaryotes, likely reflecting mutualism, commensalism or competition, could play a stabilizing role of microbial plankton communities in this productive ecosystem. Despite some limitations in the temporal coverage, the results reported here contribute to better understand the factors modulating phytoplankton succession and highlight the role of bacteria as pivotal elements. Further laboratory co-culture experiments are needed to demonstrate the biotic interactions predicted by the co-occurrence analyses.

## Supplementary Information

Below is the link to the electronic supplementary material.

Supplementary file1 (PDF 1.13 MB)

## Data Availability

Not applicable.
