# Supplementary material for: Role of Bacterial Community Composition as a Driver of the Small-Sized Phytoplankton Community Structure in a Productive Coastal System
Source: Microb Ecol. 2022 Oct 28;86(2):777–94. doi: 10.1007/s00248-022-02125-2 (PMC10335964; doi:10.1007/s00248-022-02125-2)
Supplement: Supplementary file 1 — Supplementary file1 (PDF 1.13 MB) [file 248_2022_2125_MOESM1_ESM.pdf]

Role of bacterial community composition as a driver of the small-sized phytoplankton community structure in a productive coastal system

Cecilia Costas-Selas[1*], Sandra Martínez-García[1], Ramiro Logares[2], Marta Hernández-Ruiz[1], Eva Teira[1]

[1*] Centro de Investigación Mariña, Universidade de Vigo, Departamento de Ecoloxía e Bioloxía Animal, 36310 Vigo, Spain

[2] Departament de Biologia Marina i Oceanografia, Institut de Ciéncies del Mar (ICM), CSIC, Barcelona, Catalonia, Spain.

[*] Corresponding author. E-mail: cecilia.costas.selas@uvigo.es

Journal: Microbial Ecology

SURFACE			30 METERS		
**Month**	**OTUs**	**Shannon Index**	**Month**	**OTUs**	**Shannon Index**
**Jan.14**	689	5.549	**Jan.14**	407	5.154
**Feb.14**	618	5.273	**Feb.14**	689	5.736
**Mar.14**	593	5.252	**Mar.14**	751	5.715
**Apr.14**	594	5.399	**Apr.14**	554	4.975
**May.14**	614	5.253	**May.14**	727	5.611
**Jun.14**	547	5.075	**Jun.14**	579	5.156
**Jul.14**	NA	NA	**Jul.14**	NA	NA
**Aug.14**	NA	NA	**Aug.14**	NA	NA
**Sep.14**	622	5.309	**Sep.14**	536	5.079
**Oct.14**	757	5.458	**Oct.14**	638	5.367
**Nov.14**	841	5.666	**Nov.14**	818	5.753
**Dec.14**	795	5.657	**Dec.14**	815	5.712
**Jan.15**	739	5.566	**Jan.15**	901	5.892
**Feb.15**	969	6.005	**Feb.15**	875	5.934
**Mar.15**	583	5.058	**Mar.15**	729	5.555
**Apr.15**	364	3.758	**Apr.15**	490	3.952
**May.15**	543	5.104	**May.15**	459	4.678
**Jun.15**	491	4.673	**Jun.15**	590	4.868
**Jul.15**	623	5.308	**Jul.15**	571	5.061
**Aug.15**	453	4.638	**Aug.15**	696	5.271
**Sep.15**	550	5.084	**Sep.15**	767	5.568
**Oct.15**	693	5.284	**Oct.15**	762	5.546
**Nov.15**	745	5.526	**Nov.15**	752	5.654
**Dec.15**	NA	NA	**Dec.15**	NA	NA
**Average ± SD**	639±138	5.233±0.463	**Average ± SD**	671±137.874	5.345±0.475

Table S1 Number of reads and diversity indices (Richness and Shannon index) in each sample after subsampled OTUs of bacteria.

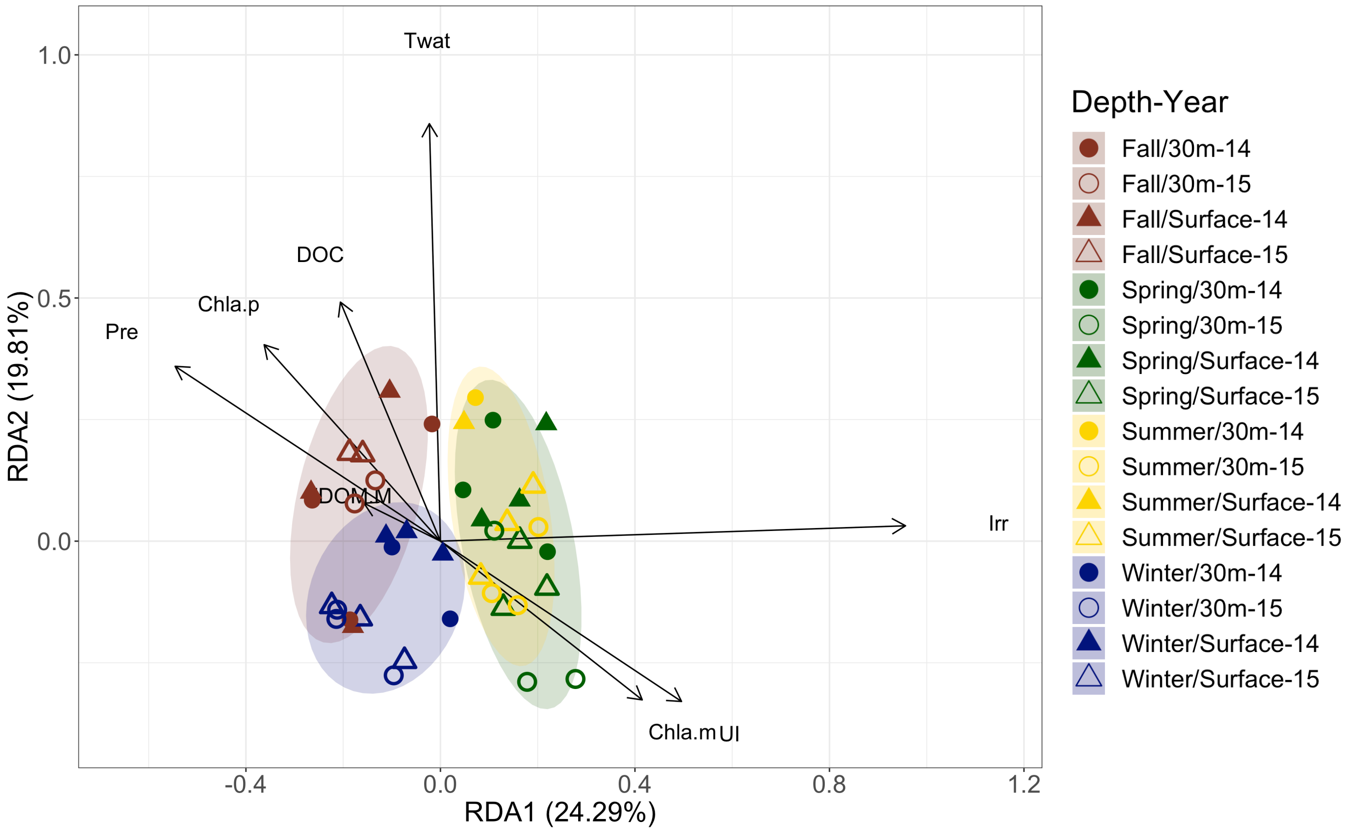

Fig. S1 Redundancy analysis (RDA) of small size-fraction (0.2–20 μm cell-size) eukaryotic community. Filled and open symbols represent samples from 2014 and 2015, respectively. Circles represent samples from 30 meters and triangles from surface. Coloured ellipses highlight the divergence of the samples in summer (yellow), fall (brown), winter (blue) and spring (green). The arrows represent the significant variables that explained variability in the structure of the community. Abbreviations: Twat (temperature of water), DOC (dissolved organic carbon), FDOM.M (humic-like dissolved organic matter fluorescence), Irr (solar radiation), UI (upwelling index), Pre (precipitation), Chla.p (pico-plankton chlorophyll-*a* and Chla.m (micro-sized chlorophyll-*a*).

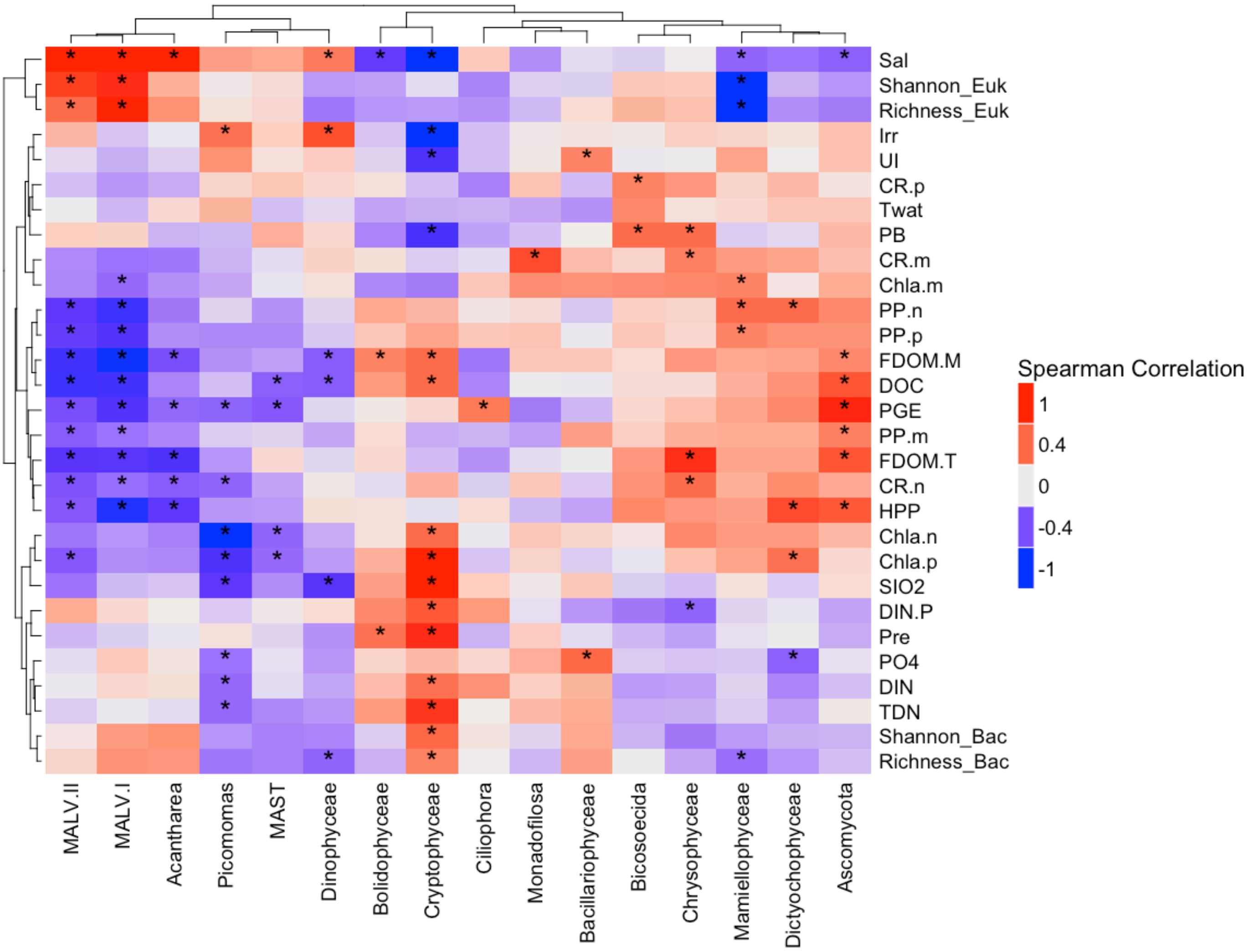

Fig. S2 Spearman correlation of the small size-fraction (0.2–20 µm cell-size) eukaryotes with abiotic and biotic variables: salinity (Sal); solar radiation (Irr); temperature of water (Twat); upwelling index (UI); dissolved inorganic nitrogen and phosphate ratio (DIN.P); precipitation (Pre); eukaryote and bacteria Shannon index (Shannon_euk and Shannon_bac); phosphate ($P0_4$); humic-like dissolved organic matter fluorescence (FDOM.M); protein-like dissolved organic matter fluorescence (FDOM.T); silicate (SIO_2); dissolved total nitrogen (TDN); dissolved inorganic nitrogen (DIN); pico-, nano- and micro-sized chlorophyll-*a* (Chla.p, Chla.n and Chla.m); pico-, nano- and micro-sized community respiration (CR.p, CR.n and CR.m); prokaryote biomass (PB); heterotrophic prokaryote production (HPP); prokaryotic growth efficiency (PGE); pico-, nano- and micro-sized primary production (PP.p, PP.n and PP.m) and dissolved organic carbon (DOC). Dendrograms represent clustering of bacteria based on their correlations with abiotic and biotic variables (Euclidean distance). Asterisks symbolism the significant correlations ($p < 0.05$).

EUKARYOTE	NEIGHBORHOOD CONNECTIVITY	DEGREE
**Dinophyceae_12**	2.5	6
**Geminigera cryophila_2**	2.5	8
**Strombidiidae**	2.9	8
**Dinophyceae_8**	3.4	8
**Micromonas pusilla_2**	3	3
**MALV-III_2**	3	11
**MALV-II_1**	2.7	3
**MALV-I_2**	6	1
**MALV-II_2**	1.7	3
**Dinophyceae_10**	2.9	9
**MALV-I_3**	6	1
**Skeletonema grethae**	3	1
**MALV-I_5**	1	1
**Minidiscus trioculatus_3**	2	2
**Chaetoceros sp.**	2	1
**MALV-II_3**	2	2
**Ostreococcus tauri**	4.3	3
**Pseudo-nitzchia sp.**	3.7	6
**MALV-III_1**	4	2
**Prorocentrum minimum_3**	6	1
**Picobiliphyta sp.**	2.8	4
**Dinophyceae_6**	2	1
**Geminigera cryophila_1**	4.7	3
**Bathycoccus prasinos**	3	4
**Average ± SD**	3.2±1.4	3.8±3.0

Table S2 Neighborhood connectivity and degree of each eukaryotic OTU from the small size-fraction (0.2–20 μm cell-size) eukaryote and free-living bacteria (< 3 μm size fraction) network with HF correction.

BACTERIA	NEIGHBORHOOD CONNECTIVITY	DEGREE
**SAR406_clade_2**	5.7	3
**Synechococcus sp._1**	6	2
**NS5_marine_group_1**	9.5	2
**Synechococcus sp._2**	4.7	3
**Amylibacter_1**	7	5
**Amylibacter_2**	6	3
**Planktomarina sp._2**	6.3	3
**SAR406_clade_1**	4.8	5
**Flavobacterium sp._2**	8	1
**Planktomarina sp._1**	6.3	3
**ZD0417_marine_group**	2.5	6
**Owenweeksia_3**	2	3
**SAR11_clade_4**	7.2	6
**NS5_marine_group_2**	8	1
**Flavobacterium sp._3**	8	1
**SAR11_clade_8**	8.7	3
**OM1_clade_1**	9.5	2
**SAR11_clade_3**	8.5	4
**OM1_clade_2**	7.7	3
**ZD0405_2**	3	1
**NS4_marine_group_2**	6	2
**SAR86_clade_3**	5	2
**Flavobacterium sp._1**	8	1
**SAR11_clade_12**	8.7	3
**Owenweeksia_1**	6	2
**NS2b_marine_group**	1	1
**ZD0405_3**	3	1
**Roseovarius sp.**	3.5	2
**SAR11_clade_5**	11	1
**SAR11_clade_2**	2	1
**Owenweeksia_2**	9	1
**SAR11_clade_9**	10	2
**SAR11_clade_13**	3	1
**OCS116_clade_2**	5	2
**SAR11_clade_7**	10	2
**SAR11_clade_11**	3	1
**OCS116_clade_1**	6	1
**NS9_marine_group_1**	8	1
**NS9_marine_group_2**	4.5	2
**SAR86_clade_2**	2	1
**SAR86_clade_4**	4	1
**Ascidiaceihabitans**	6	1
**Average ± SD**	6.1±2.6	2.2±1.4

Table S3 Neighborhood connectivity and degree of each bacterial OTU from the small size-fraction (0.2–20 µm cell-size) eukaryote and free-living bacteria (< 3 µm size fraction) network with HF correction.
